# Do general practice management and/or team care arrangements reduce avoidable hospitalisations in Central and Eastern Sydney, Australia?

**DOI:** 10.1186/s12913-019-4663-3

**Published:** 2019-11-07

**Authors:** Heidi Welberry, Margo Linn Barr, Elizabeth J. Comino, Ben F. Harris-Roxas, Elizabeth Harris, Shona Dutton, Tony Jackson, Debra Donnelly, Mark Fort Harris

**Affiliations:** 1 Centre for Primary Health Care and Equity, Faculty of Medicine, University of New South Wales, Level 3, AGSM Building, Sydney, NSW 2052 Australia; 2 Health Equity Research and Development Unit, Sydney Local Health District, NSW, Level 9, King George V Building, PO Box 374, Camperdown, NSW 2050 Australia; 3 The Royal Australian & New Zealand College of Radiologists, Level 9, 51 Druitt Street, Sydney, NSW 2000 Australia; 4 Central and Eastern Sydney Primary Health Network, Tower A, L5, 201 Coward Street, Mascot, NSW 2020 Australia; 5 South Eastern Sydney Local Health District, NSW Health, District Executive Unit, Locked Mail Bag 21, Tarren Point, NSW 2229 Australia; 6 Sydney Local Health District, NSW Health, Level 11, KGV Building, Missenden Road, Camperdown, NSW 2050 Australia

**Keywords:** Integrated care, Care plan, Coordinated care, Hospitalisations, Primary health care

## Abstract

**Background:**

The number of people living with chronic health conditions is increasing in Australia. The Chronic Disease Management program was introduced to Medicare Benefits Schedule (MBS) to provide a more structured approach to managing patients with chronic conditions and complex care needs. The program supports General Practitioners (GP)s claiming for up to one general practice management plan (GPMP) and one team care arrangement (TCA) every year and the patient claiming for up to five private allied health visits. We describe the profile of participants who claimed for GPMPs and/or TCAs in Central and Eastern Sydney (CES) and explore if GPMPs and/or TCAs are associated with fewer emergency hospitalisations (EH)s or potentially preventable hospitalisations (PPH)s over the following 5 years.

**Methods:**

This research used the CES Primary and Community Health Cohort/Linkage Resource (CES-P&CH) based on the 45 and Up Study to identify a community-dwelling population in the CES region. There were 30,645 participants recruited within the CES area at baseline. The CES-P&CH includes 45 and Up Study questionnaire data linked to MBS data for the period 2006–2014. It also includes data from the Admitted Patient Data Collection, Emergency Department Data Collection and Deaths Registry linked by the NSW Centre for Health Record Linkage.

**Results:**

Within a two-year health service utilisation baseline period 22% (5771) of CES participants had at least one claim for a GPMP and/or TCA. Having at least one claim for a GPMP and/or TCA was closely related to the socio-demographic and health needs of participants with higher EHs and PPHs in the 5 years that followed. However, after controlling for confounding factors such as socio-demographic need, health risk, health status and health care utilization no significant difference was found between having claimed for a GPMP and/or TCA during the two-year health service utilisation baseline period and EHs or PPHs in the subsequent 5 years.

**Conclusions:**

The use of GPMPs and/or TCAs in the CES area appears well-targeted towards those with chronic and complex care needs. There was no evidence to suggest that the use of GPMPs and /or TCAs has prevented hospitalisations in the CES region.

## Background

The number of people, particularly older people living with chronic health conditions and disability, is increasing in the Australian population [1]. Health service providers are grappling with the increased burden on their services due to the ongoing demands of managing these conditions that frequently have complex care needs involving multiple health care providers in both the hospital and community settings [2]. Key to the development of care for these people is ensuring access to coordinated, integrated, cost effective services that are tailored to the needs of users and providers [3, 4]. An important component of service development is improving primary prevention and wellness programs to reduce the need for acute care services particularly unplanned hospitalisations [2, 5]. Health care reforms in recent years through the restructure of public health services to Local Health Networks or Districts (LHDs/LHNs, terminology varies by jurisdiction) and the formation of Primary Health Networks (PHNs), include provision of better integrated and coordinated health care as key components of their health care strategies [2, 6].

Ensuring primary and community-based services are well placed to deliver this care has required changes in the ways that these services are provided, particularly in changes from episodic to ongoing care [7]. To recognise and facilitate the effort required by General practitioners (GP) in planning ongoing care and coordinating a treatment team, the Enhanced Primary Care package was introduced into the Medicare Benefits Schedule (MBS) in 1999 with specific item numbers for GPs to undertake care planning and coordinate team care arrangements for patient with a chronic condition or terminal medical condition [8]. This scheme evolved into the Chronic Disease Management (CDM) program in 2005 and additional items have been added to support the inclusion of services provided by private allied health providers (2004) and nurse practitioners (2007) [9, 10].

This program supports GPs to claim for a maximum of one general practice management plan (GPMP) preparation and one team care arrangement (TCA) every 12 months. The recommendation is to prepare a new plan/TCA every 2 years with review points at 6, 12 and 18 months [11]. The patient can additionally claim for up to five services provided by private allied health providers and practice nurses (from 2007 onwards) within each calendar year. The addition of support for private allied health care in particular may improve the equity of access to this type of care – previously this would only have been available to those with private health insurance ‘extras’ cover or at considerable out of pocket cost [9].

Through promoting planning of care, ongoing monitoring of complex conditions, and supporting additional allied health care, this suite of items within the MBS has the potential to positively impact patients with chronic conditions through better symptom control and prevention of disease progression or complications arising. The anticipated flow-on effect would be to maintain the provision of care in the community setting for a longer time period and reduce the likelihood of more expensive hospitalisations – a benefit to the patient as well as the health care system [7]. The use of these items has been found to be limited though, and the barriers to their use by both patients and GPs remain unclear [12].

Although previous research has been undertaken to describe the characteristics of the 45 and Up Study participants who had a GPMP and/or TCA claim prior to recruitment [13] they did not examine if there was a relationship between claims for a GPMP and/or TCA and subsequent hospitalisation. Vitry et al. [14] and Caughey et al. [15] did examine the association between GPMPs within Australia and found that GPMPs were associated with reduced hospitalisations. However, their studies were limited to Australian war veterans aged 65 years and over and to veterans with congestive heart failure [14] and diabetes [15].

The aim of this research was to examine rates of utilisation of GPMPs or TCAs for cohort participants within the CES area over the period 2006–2014. As most participants (75%) who had a GPMP also had a TCA and nearly all (97%) of those who had a TCA had a GPMP these items were grouped together as one measure “GPMP and/or TCA” based on whether there was a claim for either one or both of the items in that period. Specifically, the research sought to answer the following: (i) What proportion of participants, within the CES area, had at least one GPMP and/or TCA claim? (ii) What participant characteristics are associated with having at least one GPMP and/or TCA claim? and (iii) Do participants for whom there is at least one GPMP and/or TCA claim have reduced EHs and/or PPHs?

## Methods

### Sample

This research used the newly established CES Primary and Community Health Cohort/Linkage Resource (CES-P&CH) based on the Sax Institute’s 45 and Up Study to identify a community-dwelling population in CES to be used to answer policy relevant research questions. There were 30,645 participants recruited within the CES area at baseline. The CES-P&CH includes 45 and Up Study questionnaire data linked to MBS data for the period 2006–2014 by the Sax Institute using a unique identifier. It also includes data from the Admitted Patient Data Collection (APDC), Emergency Department Data Collection and Deaths Registry linked by the NSW Centre for Health Record Linkage (CHeReL) using probabilistic techniques [16]. CES-P&CH, based on the 45 and Up Study, was used to answer the research questions because it contained demographic and health behaviour data linked to MBS, hospitalisations and death administrative data.

All 45 and Up Study participants aged 45 years and over who were recruited within CES were eligible for the study. This target population was selected because Douglas et al. [13] found that GPMPs and/or TCAs although increasing with age were 13% overall in people aged 45–59 year and up to 35% for chronic conditions such as diabetes.

Participants were excluded from the study if they were recruited prior to 2007 (due to insufficient data for all MBS variables required) or if possible data linkage errors were identified or missing/out of range data were present on key variables such as recruitment date or age. There were 26,291 participants remaining in the sample.

### The 45 and Up Study

The 45 and Up Study comprises more than 250,000 residents of NSW, Australia. Details of the recruitment of this cohort have been described previously [17]. Potential Study participants aged 45 years or older in NSW were randomly sampled from the Department of Human Services enrolment database. They were sent an invitation to participate, a description of the Study, a self-administered questionnaire, and a consent form. Participants joined the Study by completing the baseline questionnaire and providing consent for long-term follow up, including linkage of their questionnaire data to health records being collected by public health authorities. Recruitment occurred between 2006 and 2009, with 70% of the sample being recruited in 2008. The baseline questionnaire collected information on a range of participant characteristics [18]. The response rate was 18%. MBS data were supplied by the Australian Government Department of Human Services and deterministically linked to the 45 and Up Study participants using a unique identifier. The remaining datasets were probabilistically linked by the NSW Centre for Health Record Linkage, with quality audits showing fewer than 0.5% false positive links.

### Measures

Participant characteristics were grouped into four main categories: socio-demographic; health risk factors; health status; and health care utilisation. Table 1 provides the definitions of these variables. A health service utilisation baseline period was defined for calculating health care utilisation as the two-year window centred around a participant’s recruitment date to the 45 and Up Study, referred to as the ‘*baseline service period’*.

**Table 1:** Participant characteristics – definitions and data sources

Domain	Characteristic	Data source	Description
Socio-Demographic	Age group	45 and Up Study Baseline	Self-reported age at baseline
	Gender	45 and Up Study Baseline	Self-reported sex
	Language other than English	45 and Up Study Baseline	Whether a participant speaks a language other than English at home (yes or no)
	Country of birth	45 and Up Study Baseline	Self-reported country of birth categorised as Australia or overseas
	Highest qualification	45 and Up Study Baseline	Self-reported highest level of educational qualification – categorised as less than year 12; year 12; trade/diploma; university or higher
	Household income	45 and Up Study Baseline	Self-reported household income category
	Work status	45 and Up Study Baseline	Working status at baseline: not working; working part-time; working full-time
	Housing type	45 and Up Study Baseline	Current housing type grouped as: house; flat/unit; nursing home/ residential aged care; other (including mobile home)
	Private health insurance	45 and Up Study Baseline	Private health status at baseline, grouped as: none (no private health, DVA or health care card; private health with extras; private health without extras; DVA only; health care card only
Health Risk Factor	Smoking status	45 and Up Study Baseline	Smoking status at baseline: non-smoker; ex-smoker; current smoker
	Adequate physical activity	45 and Up Study Baseline	Based on the amount of moderate and vigorous exercise reported: yes (adequate) – see AIHW definition; no (not adequate)
	Adequate fruit and vegetable consumption	45 and Up Study Baseline	Based on self-reported fruit and vegetable consumption; yes (adequate) – at least 5 serves of vegetables and 2 serves of fruit; no (not adequate)
	Weekly alcohol intake	45 and Up Study Baseline	Based on self-reported number of standard drinks each week, categorised as zero; low (<=14 drinks per week); high (> 14 drinks per week)
	Body Mass Index (BMI) category	45 and Up Study Baseline	Based on self-reported height and weight. Categorised as underweight (< 20); normal weight (20–25); overweight (25–30); obese (> 30)
	Treatment for high blood pressure	45 and Up Study Baseline	Self-reported as currently taking treatment for high blood pressure (yes or no)
	Treatment for high cholesterol	45 and Up Study Baseline	Self-reported as currently taking treatment for high cholesterol (yes or no)
Health Status	Physical functioning	45 and Up Study Baseline	Based on the Short Form 36 (SF36) standard categories
	Psychological distress	45 and Up Study Baseline	Based on the Kessler 10 (K10) standard categories
	Self-rated good/very good health	45 and Up Study Baseline	Based on the SF1 – classified as yes if responded as good, very good or excellent
	Self-rated good/very good quality of life	45 and Up Study Baseline	Based on self-rated quality of life question – classified as yes if responded as good; very good or excellent
	Number of chronic conditions	45 and Up Study Baseline	Based on self-reported diagnoses for up to six chronic diseases. These conditions were classified as: diabetes; cardiovascular disease; depression/anxiety; musculoskeletal (arthritis and osteoarthritis); asthma; and cancer.
	Needs help for a disability	45 and Up Study Baseline	Do you regularly need help with daily tasks because of long-term illness or disability? (yes or no)
	Reported a fall in the last 12 months	45 and Up Study Baseline	Self-reported (yes or no)
Health care utilisation	Average number of GP visits per annum	MBS	Calculated across a 2-year period +/−1 year from date of recruitment. Only standard GP consultations included.
	Continuity of care with - provider	MBS	Calculated across a 2-year period +/−1 year from date of recruitment. Only standard GP consultations included. Based on the Usual Provider Index (UPI) using scrambled provider number – a participant was classified as having continuity of care if 75% or more of their visits were with the same provider. Those with less than 4 visits within this period were classified as “infrequent GP visits”.
	Hospitalised at baseline	APDC	Calculated across a 2-year period +/−1 year from date of recruitment. Classified as “yes” if any hospitalisation in this period.
	Saw a specialist at baseline	MBS	Calculated across a 2-year period +/−1 year from date of recruitment. Classified as “yes” if any specialist item in this period.

The exposure measures were claims for GPMP and TCA. GPMP and/or TCA was defined as a dichotomous variable with a response of “yes” for those that had at least one claim for GPMP (Item No. 723) or TCA (Item No. 725) within the *baseline service period* and “no” if no claims for these items occurred within the *baseline service period*.

The outcome measures were emergency department visit that led to a hospital admission—emergency hospitalisation (EH) and potentially preventable hospitalisations (PPH). These were used to represent unplanned hospital admissions or hospital admissions that could potentially have been avoided through proactive management of chronic conditions. PPHs are defined as admissions to hospital that could have potentially been prevented through the provision of appropriate non-hospital health services according to the PPH indicator in the Australian 2012 National Healthcare Agreement [19]. This indicator was composed of admissions for 21 conditions, broadly categorized as “chronic,” “acute,” and “vaccine-preventable”. The conditions defined as “chronic” included as a principal diagnosis: asthma, congestive cardiac failure, diabetes complications, Chronic obstructive pulmonary disease, angina, iron deficiency anaemia, hypertension, nutritional deficiencies, rheumatic heart disease. Those defined as “acute” included as a principal diagnosis: dehydration and gastroenteritis, pyelonephritis, perforated/bleeding ulcer, cellulitis, pelvic inflammatory disease, ear, nose and throat infections, dental conditions, appendicitis with generalised peritonitis, convulsions and epilepsy, and gangrene. Those defined as “vaccine-preventable” included: influenza and pneumonia, and other vaccine-preventable conditions. These PPHs are very similar to the ambulatory care-sensitive conditions as specified in the National Health Service Outcomes Framework [20].

### Statistical analyses

The statistical analysis included three components (i) a descriptive analysis to calculate the proportion of participants within the CES area for whom there was at least one GPMP and/or TCA claim and the characteristics of those with GPMP and/or TCA claims; (ii) logistic regression to examine which factors were significantly related to at least one GPMP and/or TCA independently of the other factors and (iii) a time to event linkage analysis to examine if having had at least one GPMP and/or TCA claim at baseline reduce EHs and/or PPHs in the subsequent 5 years.

The descriptive analysis included information captured at baseline: either in the baseline 45 and Up Study survey or within the *baseline service period* (+/− 12 months from date of recruitment to the 45 and Up Study). Descriptive analyses were undertaken to examine the proportion of people with at least one GPMP and/or TCA claim by each socio-demographic, health risk factor, health status and health service utilisation characteristic of interest.

The logistic regression was then used to examine which factors were significantly related to having at least one GPMP and/or TCA independently of the other factors. All factors were included in the model.

**Fig. 1:**
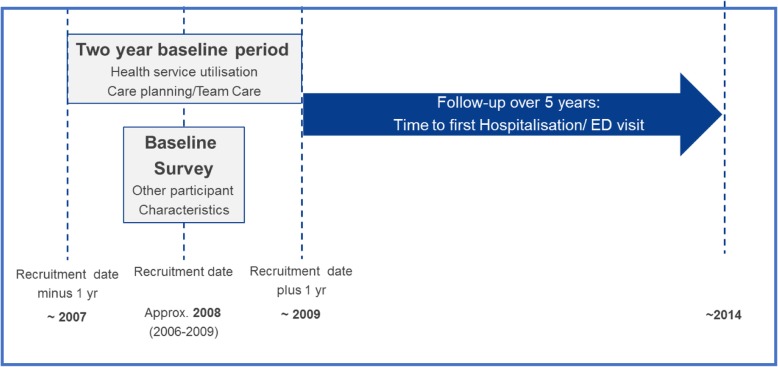
Research project design – factors associated with time to hospitalisation/emergency department (ED) visit. Description: Diagram of the research project design, specifically showing the baseline period and the follow-up period

Time to event linkage analysis included information captured at baseline: either in the baseline 45 and Up Study survey or within the *baseline service period* (+/− 1 year from date of recruitment to the 45 and Up Study) and hospital/ ED admissions in the five-year period starting from the end of the *baseline service period* (+ 1 year from recruitment). Figure 1 summarisesthe approach taken for this analysis.

Two outcomes were investigated: EHs and PPHs. Outcomes were censored at first hospitalisation, death or 5 years following recruitment date, whichever occurred first. Cox proportional hazards regression modelling was used to examine the relationship between the MBS measures and these outcome variables, controlling for all socio-demographic, health risk factor, health status and health care utilisation factors.

Additional sensitivity tests were undertaken by calculating the propensity of a person to claim for a GPMP or TCA and then using these propensity scores within a weighted analysis in line with the procedures recommended by Griffin et al. [21] for analysis of non-equivalent groups. Boosted regression using the “twang” package in R (and including all covariates specified in table1) was used to calculate propensity scores. Cox proportional hazards regression analysis was then undertaken using the “survey” package in R with the propensity scores included as weights.

## Results

### Use of general practice management and/or team care arrangements within the CES cohort

**Fig. 2:**
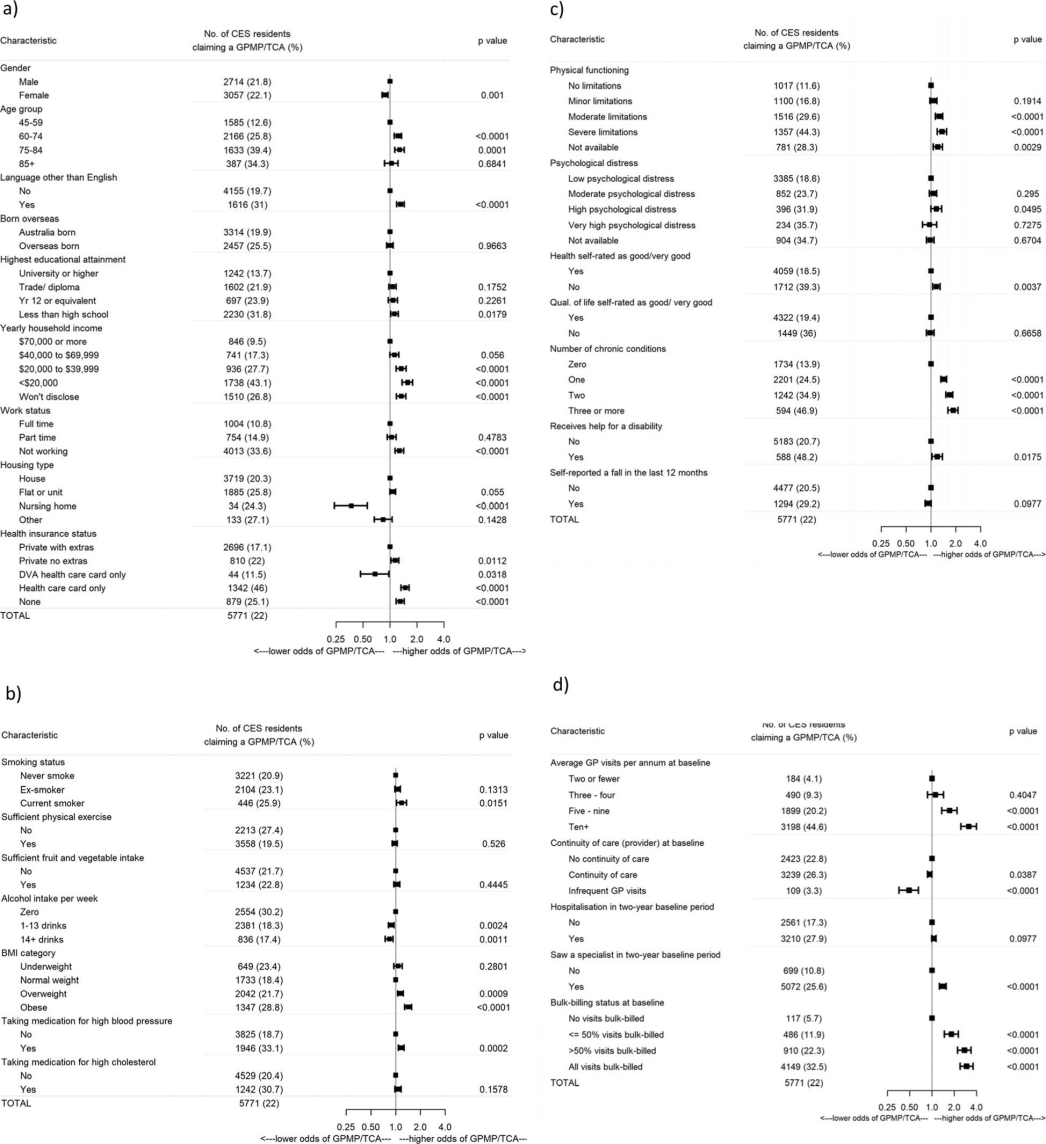
Adjusted Odds Ratios of claiming for a GPMP/TCA by (**a**) socio-demographic, (**b**) health risk, (**c**) health status and (**d**) health care utilisation factors adjusting for all other socio-demographic, health risk, health status and health care utilisation factors. Description: Forest plots showing the characteristics, numbers and percentages, odds ratios and *p*-values

Within the two-year health service utilisation *baseline service period* (approx. 2007–2009), 22% of the CES cohort (5771 people) had at least one claim recorded for a GPMP and/or TCA. As shown in Fig. 2 claims for preparation of a GPMP and/or TCA in the CES cohort were more frequent (over 30%) in: older age groups (39.4% and 34.3%); language background other than English (31.0%); lower educational attainment (31.8%); lower income (43.1%); not working (33.6%); having a health care card (46.0%); taking medication for high blood pressure (33.1%); taking medication for high cholesterol (30.7%); not drinking alcohol (30.2%); more severe physical limitations (44.3%); higher levels of psychological distress (31.9%); lower self-rated health (39.3%); lower self-rated quality of life (36.0%); three or more chronic conditions (46.9%); needed help for a disability (48.2%); more visits to the GP (44.6%); and all visits bulk-billed (32.5%).

### CES cohort characteristics associated with receiving a GPMP and/or TCA.

As shown in Fig. 2, after controlling for all other socio-demographic, health risk, well-being and health utilisation factors, higher proportions of at least one GPMP and/or TCA claim were associated with: speaking a language other than English, having a lower income, having a lower educational attainment, being 60–84 years, not working, having a health care card, not having private health insurance, being a current smoker, having moderate/severe physical limitations, having fair or poor self-rated health status, having one or more chronic conditions, needing help for a disability, having five or more GP visits, seeing a specialist in the *baseline service period*, and having bulk-billed visits.

Also as shown in Fig. 2, after controlling for all other socio-demographic, health risk, well-being and health utilisation factors, lower proportions of at least one GPMP and/or TCA claim were associated with: being female, living in a care facility, having a DVA card, drinking alcohol, being overweight, being obese, being on high blood pressure medications and infrequently visiting a GP.

### Association between GPMP and/or TCA and emergency and/or potentially preventable hospitalisations.

In the five-year period following recruitment 7323 CES cohort participants had an EH and 3468 had a PPH. CES cohort participants with at least one GPMP and/or TCA claim in the *baseline service period* had higher rates of EHs (43.2% v 23.5%) and PPH (21.6% v 10.8%) during this 5-year period.

As shown in Table 2 after controlling for confounding factors such as socio-demographic, health risk, health status and health care utilization no significant difference was found between having claimed for a GPMP and/or TCA in the *baseline service period* and EHs (AdjHR = 1.06, 95%CI: 1.00–1.12) or PPHs (AdjHR = 1.05, 95%CI: 0.98–1.14) in the subsequent 5 years. In the sensitivity testing, using the propensity analyses, there was also no significant difference was found between having claimed for a GPMP and/or TCA in the *baseline service period* and EHs (AdjHR = 1.04, 95%CI: 0.98–1.10) or PPHs (AdjHR = 1.03, 95%CI: 0.95–1.12).

**Table 2:** Relationship between having a GPMP and/or TCA during the *baseline service period* and (i) Potentially preventable hospitalisation and (ii) Emergency hospitalisation

GPMP or TCA at baseline	n	Emergency Hospitalisation within 5 years					Potentially Preventable Hospitalisation within 5 years				
		Numbers and percentages				Adjusted Model^a^	Numbers and percentages				Adjusted Model^a^
		yes	%	no	%	HR (95% CI)	yes	%	no	%	HR (95% CI)
No	20,520	4829	23.5	15,691	76.5	1 1.06 (1.00–1.12)	2219	10.8	18,301	89.2	1 1.05 (0.98–1.14)
Yes	5771	2494	43.2	3277	56.8		1249	21.6	4522	78.4	
TOTAL	26,291	7323	27.9	18,968	72.1		3468	13.2	22,823	86.8	

^a^Adjusted for age, sex, language other than English, country of birth, household income, highest qualification, work status, private health insurance, smoking status, adequate physical activity, adequate fruit and vegetable consumption, weekly alcohol intake, Body Mass Index (BMI)category, treatment for high blood pressure, treatment for high cholesterol, physical functioning, psychological distress, self-rated good health, self-rated good quality of life, number of chronic conditions, needs help for a disability, self-reported a fall, average number of GP visits per annum over baseline period, continuity of care indictor, hospitalised over baseline, saw a specialist over baseline period

## Discussion

Within the two-year health service utilisation baseline service period (approx. 2007–2009), 22% of those residing in the CES area (5771 people) had a claim recorded for a GPMP and/or TCA. Having a claim recorded for a GPMP and/or TCA within the 45 and Up Study *baseline service period* was closely related to the socio-demographic and health need of a participant. Generally, the use of GPMP and/or TCAs appeared targeted to a group whose profile of socio-demographic and health need was consistent with those who have chronic and complex conditions. This targeted profile of claimants was similar to those found by Douglas et al., [13]. However, the current study also explored prior health utilisation factors as well as a much wider range of health status factors. This highlighted the strong relationship between high volume users of GP services and increased odds of claiming for a GPMP and/or TCA but after controlling for other factors did not find a relationship between prior hospitalisation and claims for a GPMP and/or TCA. This suggests that it may be repeated engagement with GP services rather than episodes of acute care that underlie decisions between a GP and patient to instigate GPMPs and/or TCAs. This suggests GPMPs and TCAs may be driven in general practice as a way of managing patients frequently presenting with chronic conditions rather than as a mechanism to avoid hospital. Previous research has shown that GPs use care plans as a way of organising clinical care, assisting patients in accessing allied health and as a way of educating patients to instigate health behavioural change [22]. As such, there may be different drivers to instigate such a plan dependent on a patient’s individual circumstances. It is also likely that there is variation between GPs in their propensity to use care plans dependent on their perception of their usefulness.

Participants who utilised GPMPs and/or TCAs in the *baseline service period* had higher rates of hospital admissions (EHs or PPHs) in the 5 years that followed. However, after controlling for confounding factors such as socio-demographic need, health risk, health status and health care utilization no significant difference was found between having claimed for a GPMP or TCA during the *baseline service period* and time to first EH and/or PPH in the subsequent 5 years.

The current study found no evidence that GPMP and/or TCAs by themselves were associated with a reduction in unplanned hospital admissions, in contrast to the associations found by Vitry et al. [18] and Caughey et al. [15]. This study examined a much more heterogeneous population both in terms of a mix of health status and chronic disease as well as not being limited to a population of Australian War Veterans. Given that DVA health insurance provides different coverage to that of the MBS – most notably DVA Gold Cards include cover for certain allied health services – it is possible that CDM items are used differently by those with DVA cover. The strength of this current study is that the sample reflects the general population living in the CES area and therefore shows the overall usage and impact of CDM items in a real-world setting. However, it is possible that the very heterogeneous nature of the population meant that CDM use was still confounded with health status diluting any underlying protective effect of GPMPs and/or TCAs. We addressed this possibility by controlling for as many aspects of health status as available. The current findings are consistent with those found by Comino et al. [23] which also used the 45 and Up Study and found that amongst those with diabetes, having a GPMP prepared was not significantly associated with a reduction in the number of hospitalisations experienced.

Further research to examine in more detail the specifics of the GPMP and/or TCAs, for example services provided as part of the plans and reasons for the hospitalisations, use of plans by GPs and patients, quality of the plans, may provide us with a better understanding of their effect. Ideally as suggested by Knott et al. [24] complementing the MBS and existing questionnaire data with information from clinicians and participants would provide a full picture to assess if the CDM program is really delaying hospitalisations and reducing the burden on the patients and the system.

## Conclusions

This is the first study that has looked at the use of GPMPs and/or TCAs in a very large general population group within Australia and the first to assess the association between GPMP and/or TCA use and measures of health care utilisation in addition to health status. The use of GPMPs and TCAs in the CES region appears well-targeted towards a group which fits the profile of those with chronic and complex care needs. Repeated engagement with GP services rather than episodes of acute care appeared to underlie the instigation of GPMPs and/or TCAs. There was no evidence found in this study to suggest that the use of GPMPs and/or TCAs has prevented hospitalisations. The reasons for this are unclear, however it is possible that a protective effect may exist for GPMPs and/or TCAs for specific sub-groups or those with particular conditions. GPMPs and/or TCAs may assist GPs in providing structured management of frequently presenting patients.

## Data Availability

The data that support the findings of this study are available from the Sax Institute, but restrictions apply to the availability of these data, which were used under license for the current study, and so are not publicly available. Data are however available from the authors upon reasonable request and with permission of the Sax Institute.

## Abbreviations

AIHW: Australian Institute of Health and Welfare
APDC: Admitted Patient Data Collection
BMI: Body Mass Index
CDM: Chronic Disease Management (formerly Enhanced Primary Care or EPC)
CES: Central and Eastern Sydney
CESPHN: Central and Eastern Sydney Primary Health Network
CES-P&CH: Central and Eastern Sydney Primary and Community Health Cohort/Resource
CHeReL: Centre for Health Record Linkage
DVA: Department of Veterans’ Affairs
ED: Emergency Department
EH: Emergency Hospitalisation
GP: General Practitioner
GPMP: General Practice Management Plan
K10: Kessler 10
LHD: Local Health District
MBS: Medicare Benefits Schedule
NHS: National Health Service
NSW: New South Wales
PHN: Primary Health Network
PPH: Potentially Preventable Hospitalisation
SESLHD: South Eastern Sydney Local Health District
SF36: Short Form 36
SLHD: Sydney Local Health District
TCA: Team Care Arrangement
